# Three-dimensional assessment of upper airway changes associated with mandibular positional deviations following fibula free flap reconstruction

**DOI:** 10.1007/s00784-024-05646-x

**Published:** 2024-04-11

**Authors:** Katja Leonie Schulz, Ragai Matta, Christopher-Philipp Nobis, Tobias Möst, Marco Kesting, Rainer Lutz

**Affiliations:** 1 Department of Oral and Cranio-Maxillofacial Surgery, University Hospital Erlangen, Friedrich-Alexander-University Erlangen-Nürnberg (FAU), Erlangen, Germany; 2 Department of Prosthodontics, University Hospital Erlangen, Friedrich-Alexander-University Erlangen-Nürnberg (FAU), Erlangen, Germany

**Keywords:** Upper airway anatomy, Three-dimensional evaluation, Mandibular reconstruction, Fibula, Mandible

## Abstract

**Objectives:**

Fibula free flaps (FFF) are the standard approach to mandibular reconstruction after partial resection, with the goal of restoring aesthetics and masticatory function. The graft position affects both and must be carefully selected. Correlations between sagittal positioning and upper airway anatomy are known from orthognathic surgery. This study aims to evaluate changes in mandibular position and upper airway anatomy after reconstructive surgery with FFF and corresponding correlations.

**Materials and methods:**

Mandibular position after reconstruction was evaluated using three-dimensional datasets of pre- and postoperative computed tomography scans of patients treated between 2020 and 2022. Three-dimensional measurements were performed on both condyles and the symphyseal region. Changes in upper airway volume and minimum cross-sectional area (minCSA) were analysed. Intra-rater reliability was assessed. Correlations between changes in upper airway anatomy and sagittal mandibular position were tested.

**Results:**

The analysis included 35 patients. Intra-rater reliability was good to excellent. Condylar deviations and rotations were mostly rated as small. Changes in symphyseal position were considerably greater. Median airway volume decreased in the oropharynx and hypopharynx. Posterior deviation of the symphysis was associated with a decreasing minCSA in the hypopharynx and vice versa.

**Conclusions:**

The overall accuracy of mandibular reconstructions with FFF is high, but there is room for optimization. The focus of research should be extended from masticatory to respiratory rehabilitation.

**Clinical relevance:**

Effects on respiratory function should be considered prior to graft positioning. The clinical relevance of upper airway changes within the complex rehabilitation of reconstructive surgery patients needs to be further investigated.

## Introduction

Partial mandibular resection is a common therapeutic procedure in maxillofacial surgery. The mandible is not only important for chewing but also for speech and respiratory function [1]. Therefore, after resection, an adequate reconstruction is mandatory to achieve functional rehabilitation of the patient. For this purpose, the fibula free flap (FFF) has become the standard approach [2]. Since its first description by Hidalgo in 1989 [3], the possibilities and the expectations of the postoperative results have increased. Much research is underway to optimize the surgical results in terms of accuracy and efficacy. Computer-aided design/computer-aided manufacturing (CAD/CAM) techniques, which allow digital planning of the surgery, and semi-standardized resection and cutting guides (ReconGuide), some of which can be adapted to the clinical situation during the surgery, can help to meet these demands [4, 5]. Including these, most modern surgical aids focus on the bony reconstruction. With good reason, because high accuracy of the bony reconstruction and position of dental implants in all three dimensions is necessary in order to achieve normal occlusion, mastication and aesthetics [6], an overall better functional outcome and a higher quality of life [7]. Whichever technique a surgeon chooses, the procedure is far from being simple and is even further complicated by the fact that the FFF has a limitation: it does not provide sufficient bone height to restore the original vertical extension of the mandible [8, 9]. The shape of the fibular neo-mandible is more like an atrophied jaw. Therefore, in order to avoid a Class III situation, the anterior fibula segment must often be placed posterior to the native symphysis to provide optimal conditions for a future implant-borne prosthetic restoration. However, in the field of orthognathic surgery, it is well known that mandibular setback surgery is associated with changes in the upper airway anatomy, which in turn may affect airway obstruction problems [10]. Mandibular and/or maxillomandibular advancement, on the other hand, is an effective therapeutic intervention for widening the upper airways in patients with obstructive sleep apnoea [11, 12]. The symphysis is furthermore the region of genial attachment. Resection of this region, and thus destruction of the muscular attachment, may affect hyoid and laryngeal movements [13]. Despite these well-known facts, airway modifications have not been the focus of research in the field of reconstructive surgery.

The present study aims to evaluate the outcomes after mandibular reconstructive surgery, focusing on the three-dimensional changes in (neo)mandibular position and of the upper airway anatomy. In addition to the descriptive analysis, the changes in mandibular position and upper airway anatomy will be examined for correlations.

## Materials and methods

### Patient selection

Between January 2020 and December 2022, patients undergoing mandibular reconstruction with a FFF were selected from the patient database of the Department of Oral and Cranio-Maxillofacial Surgery of the University Hospital Erlangen (Friedrich-Alexander-University Erlangen-Nürnberg, Germany). Inclusion criteria were preservation of both condyles, complete restoration of continuity, and the availability of pre- and postoperative computed tomography (CT) scans taken in the supine position and showing all relevant structures in a slice thickness < 1 mm. Data were collected on age, sex, diagnosis, dentition, extent and location of reconstruction, tumour size and location, neck dissection, and radiotherapy. Approval of the local ethics committee was given (reference number 23-438-Br).

### Data processing

Pre- and postoperative CT images of each included patient were transformed into three-dimensional Surface Tessellation Language (STL) data sets using medical segmentation software (Mimics Innovation suite 21; Materialise, Leuven, Belgium) to represent the bony structures of the head and neck and the upper airway. The STL data were further processed and analysed in three-dimensional CAD software (GOM Inspect 2019 and 2022; Carl Zeiss GOM Metrology GmbH, Braunschweig, Germany). The first steps of the analysis were conducted as proposed by van Baar et al. [14]: to eliminate errors caused by the segmentation process, a volume assessment was performed on the pre- and postoperative STL of each patient. Secondly, an XYZ orientation was created along the axes of the skull (X: anterior, Y: left-lateral, Z: cranial). On this basis, changes in the position of the mandible and the upper airway were analysed as follows.

**Fig. 1:**
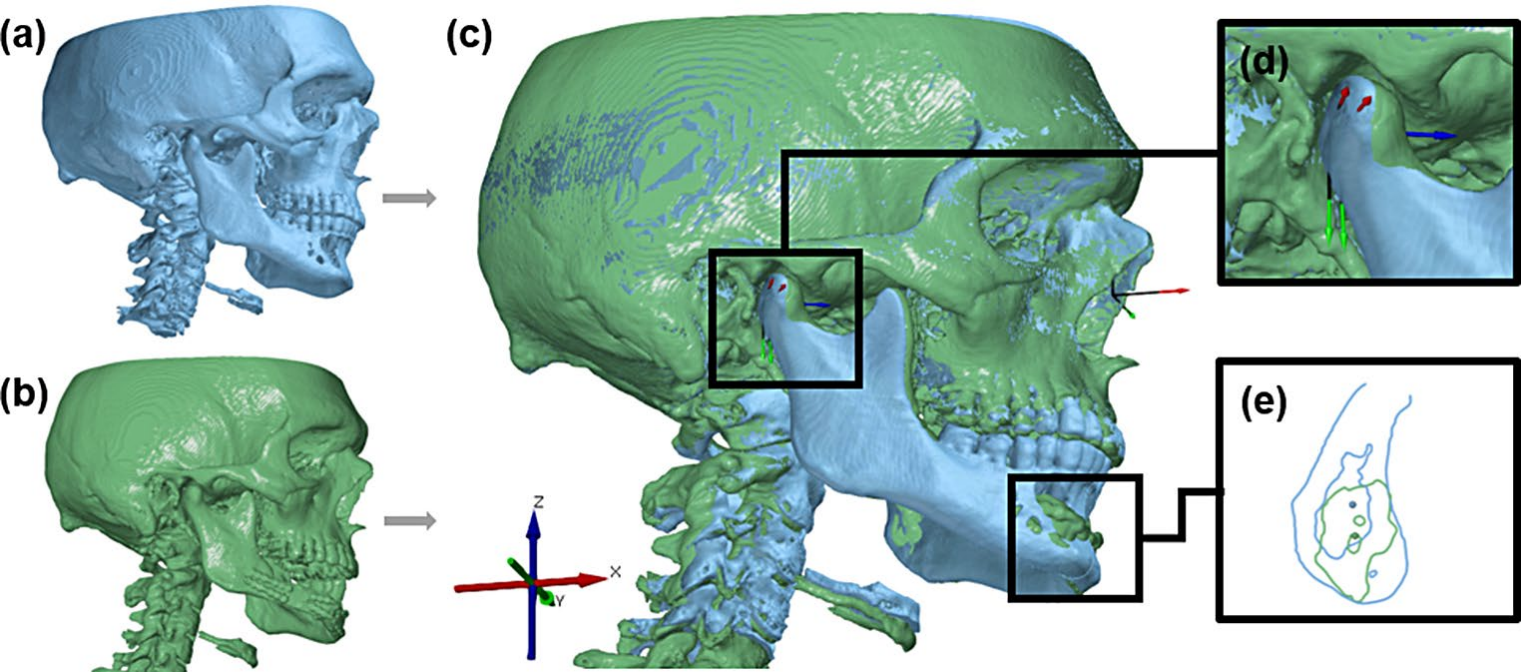
Three-dimensional measurements of the position of the (neo)mandible. Bony situation before (a) and after (b) surgery, superimposed in the area of the skull and aligned with its axes and planes (c), pre- and postoperative position of the right condyle with reference object for measurements according to Schulz et al. [15] (d), contours and centre points (S) of the symphysis region (e)

Three-dimensional positional changes of the (neo)mandible were analysed using three reference regions: the condyles and the (neo)symphysis (Fig. 1). The movements of the left (L) and right (R) condyles were evaluated using a method described by Schulz et al. [15], which allows a complete mapping of the condyles’ movement in the three-dimensional space: it determines Euclidean distances (dXYZ), deviations along the axes (dX, dY, dZ) and planes (dXY, dXZ, dYZ) of the skull and rotations around the all axes (Phi(X), Theta(Y), Psi(Z)). Unlike the condyles, the symphysis is often replaced by fibular bone, so it was necessary to apply a method to this region that allows the position of two differently shaped objects to be compared. Therefore, the intersection of the symphysis with the median sagittal plane of the skull (XZ) was constructed and its centre point (S) was calculated by the CAD software. The deviations of the centre point in the midsagittal plane (S_dXZ, S_dX, S_dZ) were then further analysed.

**Fig. 2:**
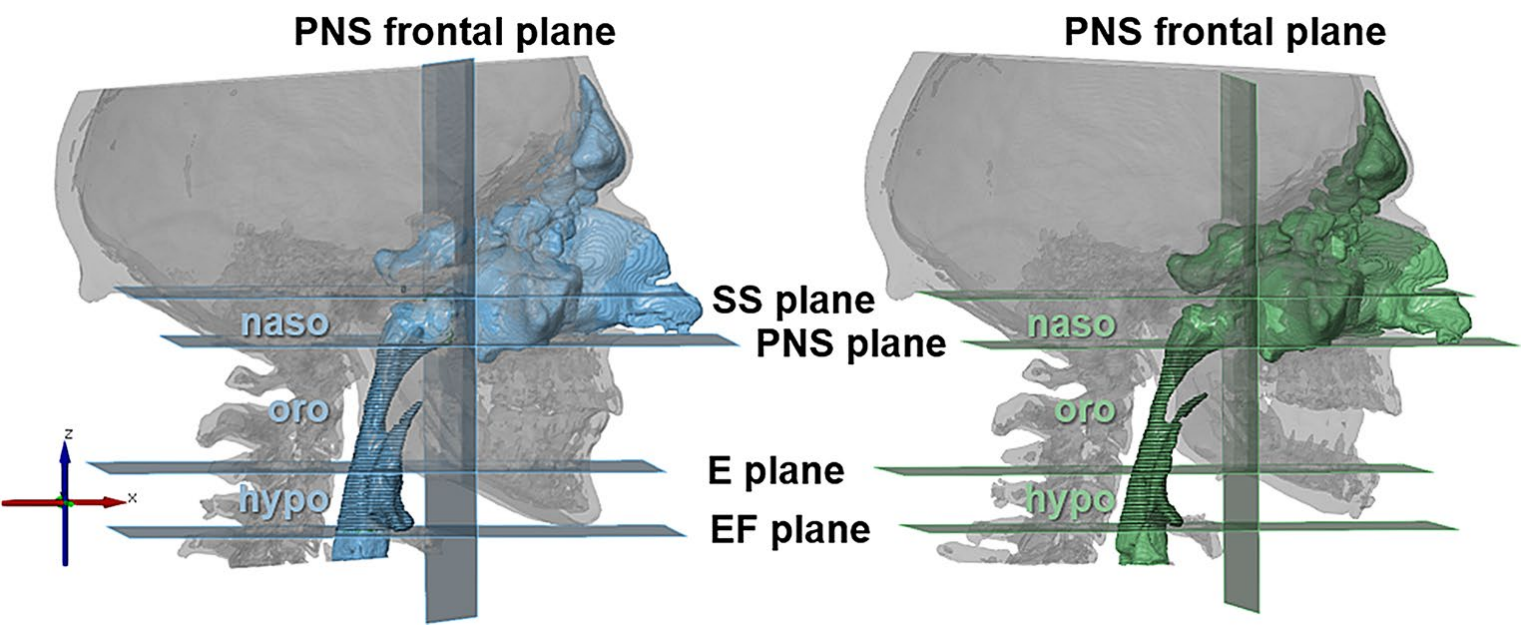
Assessment of the upper airway anatomy before (left) and after (right) surgery; reference planes defining naso-, oro- and hypopharynx according to Steegman et al. [10]. Cross-sectional areas are visualized in oro- and hypopharynx

To assess changes in the upper airway (Fig. 2), reference planes were constructed following the work of Steegman et al. [10], to define three airway segments: naso-, oro- and hypopharynx. Pre- and postoperative volumes of these segments and of all segments combined were measured and the percentage change was calculated. The cross-sectional areas of the oropharynx and hypopharynx were measured at 1 mm intervals starting from the Epiglottis plane (E plane) separating the two segments, and the minimum cross-sectional areas (minCSA) were determined.

### Statistics

First, intra-rater reliability was assessed by repeated measurements by the same observer for seven randomly selected patients (20%). Intra-class correlation coefficient (ICC) estimates and their 95% confidence intervals (CI) were calculated using a single-rating (k = 2), absolute-agreement, two-way mixed-effects model [16].

All variables were tested for normal distribution using the Shapiro-Wilk test. The demographic and clinical information of the patients was presented using descriptive statistics.

The position of the neo-mandible after reconstructive surgery was evaluated with descriptive statistics of the three-dimensional deviations and rotations of the condyles and the deviations of the (neo-)symphysis in the median-sagittal plane of the skull. Percentage changes in volume and minCSA of the upper airway were evaluated.

Next, anterior-posterior mandibular deviation (R_dX, L_dX, S_dX) was tested for correlations with changes in upper airway volume and minCSA using Spearman’s correlation coefficient. A one-tailed hypothesis test was applied: anterior mandibular deviation was expected to be associated with greater airway volume and minCSA, and posterior mandibular deviation was expected to result in smaller airway volume and minCSA.

Finally, the influence of other factors that could affect the airway (percentage changes in total and segmental volumes and of minCSA of the oropharynx and hypopharynx) was evaluated: Spearman’s correlation coefficient was calculated for metric variables (age, CT intervals) and Kruskal-Wallis (diagnosis, surgical technique, fibula segments, postoperative dentition) or Mann-Whitney-U test (sex, regions affected by malignant neoplasm, number of surgical procedures, radiotherapy, neck dissection, symphyseal resection) for non-parametric variables.

All statistical analyses were performed using statistical software (IBM SPSS Statistics 28; IBM Deutschland GmbH, Ehningen, Germany). The significance level was set at *P* = 0.05 for all analyses.

## Results

ICC estimates showed good to excellent reliability for bone measurements (average ICC: 0.992 (0.937;0.999); 17 of 23 variables with 95% CI lower bound > 0.9) and cross-sectional area measurements (average ICC: 0.989 (0.933;0.998); 3 of 4 variables with 95% CI lower bound > 0.9). Reliability was excellent for upper airway volume measurements (average ICC: 0.997 (0.976;1.000); 8 of 8 variables with 95% CI lower bound > 0.9) [16]. 

The selection process resulted in 35 eligible patients for further analyses. Reasons for exclusion were resected condyles (*n* = 2), necrosis and transplant loss prior to postoperative CT (*n* = 1), fracture of the osteosynthesis plates prior to postoperative CT (*n* = 7), missing of important regions on CTs (*n* = 5), absence of postoperative CT (*n* = 4), absence of CT prior to mandibular resection (*n* = 4) and tracheal rupture prior to postoperative CT (*n* = 1). Demographic and clinical data of the included patients are shown in Table 1. Normal distributions were reported for only eleven of the 23 variables describing mandibular movements and for none of the percentage airway measurements. Non-parametric statistics were therefore used.

**Table 1:** Patient demographic and clinical data

Patient demographic and clinical data: count (percentage)/ mean ± STD/ median (IQR).		
Patients		35 (100%)
Age in years		60 ± 10
Sex	Male	21 (60%)
	Female	14 (14%)
Diagnosis	Malignant neoplasm	17 (49%)
	Benign neoplasm	2 (6%)
	Osteonecrosis	15 (43%)
	Osteomyelitis	1 (3%)
Regions affected by malignant neoplasm (multiple options possible)	Floor of mouth	11 (31%)
	Tongue	5 (14%)
	Oropharynx	1 (3%)
Reconstruction	Primary	31 (89%)
	Secondary	4 (11%)
Surgical technique	Non-guided	7 (20%)
	CAD/CAM	4 (11%)
	ReconGuide	24 (69%)
Fibula segments	One	4 (11%)
	Two	9 (26%)
	Three	21 (60%)
	Four	1 (3%)
CT interval in days	Preoperative CT to surgery	22 (13;68)
	Surgery to Postoperative CT	164 ± 87
Radiation therapy during observation	Yes	9 (26%)
	No	26 (74%)
Neck dissection	Yes	15 (43%)
	No	20 (57%)
Resection of symphysis	Yes	32 (91%)
	No	3 (9%)
Postoperative dentition	Bilaterally supported	3 (9%)
	Unilaterally supported	9 (26%)
	No lateral support	23 (66%)

**Fig. 3:**
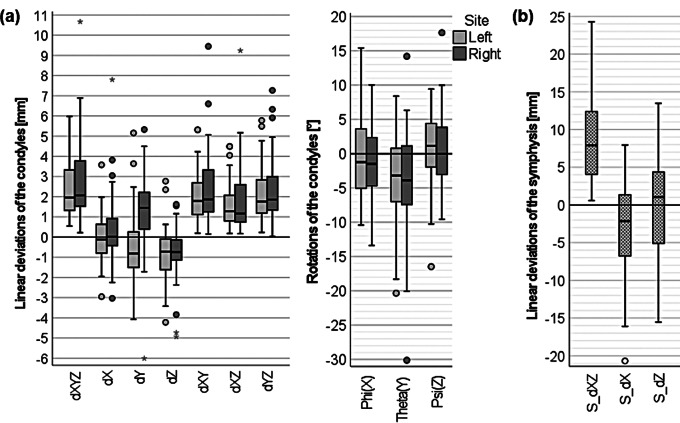
Deviations and rotations of the left and right condyles along the axes and planes of the skull (a); deviations of the symphysis along the median sagittal plane (b)

The median deviation was 2.0 (1.3;3.4) mm for the left condyle and 2.1 (1.5;3.9) mm for the right condyle. The largest deviations and rotations occurred in/along the horizontal plane and axis, the smallest in/along the sagittal plane and axis. The mean deviation of the symphysis was 7.9 (4.0;12.4) mm. The mandibular positional changes in relation to all axes and planes of the skull are illustrated in Fig. 3.

**Fig. 4:**
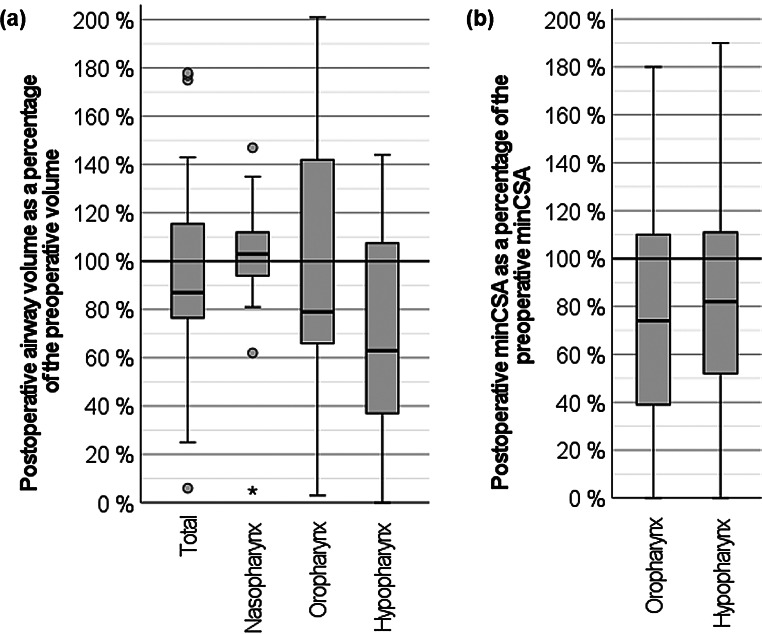
Percentage of postoperative to preoperative airway volumes (a) and minimum cross-sectional areas (b); statistical outliers above 200% are not shown for better legibility of the main results

Changes in upper airway anatomy are shown in Fig. 4. Overall, no statistically significant changes were found regarding the pre- and postoperative airway sizes. The median total airway volume was reduced to 87% (76%;116%) of the preoperative volume, though a high variance was observed. Changes in airway volume were more evident as one moved caudally. The minCSA decreased in the majority of cases.

Anterior-posterior deviation of the mandible and airway anatomy showed a significant correlation: Posterior displacement of the symphysis was associated with a decrease in the minCSA of the hypopharynx and vice versa (Spearman’s rho = 0.288, *P* = 0.047).

The other variables collected showed no clear influence on the total and segmental volumes of the upper airway, nor on its minCSA: age did not correlate with percentage airway changes; no significant differences were found for the different groups of sex, number of fibula segments, number of surgical procedures, surgical technique, symphyseal resection, neck dissection, diagnosis, malignant tumour in floor of mouth, malignant tumour in oropharynx or postoperative dentition.

The CT interval in days showed only one significant correlation (percentage change of the oropharyngeal minCSA and interval between preoperative CT and surgery (Spearman’s rho=-0.384, two-tailed *P* = 0.028)). There was a tendency for larger nasopharyngeal airway volume with malignant neoplasm not involving the tongue, compared to malignant neoplasm involving the tongue (*P* = 0.027), but no other effect. There was a trend for a smaller nasopharyngeal airway volume if the patient received radiotherapy during observation (*P* = 0.036). There was no other effect of radiotherapy.

## Discussion

According to a recent review of the literature (02/2024), this study is one of the first to evaluate correlations between airway anatomy and mandibular position after reconstructive surgery. Changes in mandibular position after microvascular fibula flap reconstruction were analysed using a method that allows three-dimensional evaluation by analysing three relevant areas: the mandibular condyles, whose position is highly relevant for mandibular movement and masticatory function [13, 17], and the symphysis, whose position is important both for later prosthodontic rehabilitation and for respiratory function, as the genioglossus muscles are attached here [1, 18]. The method proved to be highly reliable, which can be explained by several facts: all measurements were made using as much three-dimensional information as possible, the segmentation process was double-checked by volumetric measurements and the points for bone position analysis were not selected on the bony surfaces but in the centres of the regions of interest [19]. The analysis of the upper airway was performed as proposed by a meta-analysis in the field of orthognathic surgery [10] and supplemented by the measurement of the minCSA, as narrow upper airways are more prone to obstructive events [20].

Deviations and rotations of the condyles showed similar results to the previous study by the working group [15]. In most cases, the condyles’ movements were considered minor, while significant positional changes occurred in some cases. One of these more inaccurate cases might e.g. be explained by its large defect size requiring four fibula segments for reconstruction, since larger defects come with higher complexity [21]. Deviations in the position of the symphysis were greater than those of the condyles, especially in the posterior direction, indicating setback-like movements of the mandible. Matching our expectations, these setback-like movements correlated with a decreasing minCSA of the hypopharynx, which is a main finding of this study. However, no correlation was evident between mandibular setback movements and upper airway volume reduction, though both occurred simultaneously in the majority of cases. In the field of orthognathic surgery, there is huge consensus on the correlation between mandibular setback and a long-term decrease in upper airway volume and posterior airway space (PAS) [10, 22, 23]. While maxillary movement affects the nasal region, the effects of mandibular movement appear more caudally [24], which is in line with our results. Reconstructive surgery is bearing many other possible influencing factors, though, making further research mandatory before making simultaneous conclusive statements.

The limitations of this study are its retrospective design on the one hand, and the complex and multifactorial surgical procedure on the other hand. Due to the retrospective design, jaw position was not controlled during the CT scans and the time intervals between surgery and CT scans varied widely. Other factors that might have had an influence were considered in the basic analyses in this study and did not show systematic effects on the outcomes. Some significant correlations were found that we are unable to interpret logically and therefore considered them as potentially false positive findings. These type I errors may occur due to the high number of statistical tests performed. All of them are mentioned in the results section but are not further discussed to avoid frantic speculation. Furthermore, group sizes varied widely, and the brief analysis of these many influencing variables was primarily designed to rule out serious bias but would be worth a separate study, each. Though not statistically significant (*P* > 0.05), our results are e.g. suggestive of a tendentially larger airway volume if dental occlusion was preserved at least partially on both sides, than with unilateral or without occlusion. However, the preservation of dentition is highly determined by the defect size and thus difficult to evaluate separately. Previous analyses on possible risk factors for upper airway impairment after head and neck surgery have mostly been focusing on the immediate postoperative period. Cameron et al. [13] attempted to predict the need for tracheostomy after major head and neck surgery. They found that the need for a mandibulectomy and a microsurgical reconstruction were risk factors, as were tumour location and bilateral neck dissection. When performing neck dissection, the lymphatic drainage system is removed, which is associated with significant postoperative swelling [13]. However, Moubayed et al. [18] state that segmental mandibulectomy and microvascular reconstruction is possible without tracheostomy in carefully selected patients. They identified tongue and pharynx resection, defects of the central mandible – and thus genial attachment – and bilateral neck dissection as risk factors. In the present study, neither neck dissection nor tumour location showed relevant correlations with the airway anatomy. This may be due to the fact that postoperative CTs were mainly performed after the critical period of time regarding postoperative swelling, which may explain the lack of correlations and would be in consistence with a study by Du et al. [25], who also considered long-term airway changes after reconstructive surgery. Therefore, the present study explicitly does not allow the assessment of the need for tracheostomy after mandibular reconstruction. However, the measurement method combined with clinical data could be used for such an analysis in the future.

Literature on upper airway changes following reconstructive surgery is scarce and Du et al. [25] are one of the very few to describe airway changes following reconstructive surgery of the mandible. In contrast to the present study, their focus is on the influence of the tumour diagnosis rather than the (neo)mandibular position and they only included tumours of the anterior mandible. Analysing airway volumes and cross-sectional areas, their main finding was that malignant tumour resection and reconstruction resulted in widening of the upper airway, whereas this effect did not occur in benign tumours. They explain this with the deficit of soft tissue after extensive resection around a malignant tumour. In the present study, the diagnosis did not have an effect on the airways, but the size of the groups varied greatly, making it impossible to draw any conclusions. Comparison of the studies is further hampered by the fact that they analysed spiral CT scans taken with the patient in an upright position, whereas the present study evaluated only supine CT scans. The dimensions and volumes of the PAS differ depending on the patient’s position [26]. An effect on the mandible’s position, on the other hand, seems to only be apparent in the occipital-caudal axis but not in the anterior-posterior axis [26]. Mandibular movement as a function of the patient position is another potential bias that needs to be investigated in future analyses before definitive statements can be made. In contrast to orthognathic surgery, where spiral CT scans are often sufficient and where most research on this topic has been conducted to date, patients undergoing reconstructive surgery often require more extensive CT imaging. Research should therefore not be limited to analysis in the upright position, but should also extend to the supine position and to the differences between the two.

Another study addressing the influence of mandibular reconstruction on the PAS was recently published by Winnand et al. [27]. They also state that the mandible was intentionally reconstructed in a retrognathic position, when the symphysis area was involved. While this related to almost all patients of our study, they had a sufficient comparative group of cases where the central mandible had been preserved. This may be explained by the fact that they did not limit their data to fibula flaps, which are commonly used for larger defect sizes, but also included deep circumflex iliac artery flaps used for smaller defects. One of their main results depicts a decrease in the cross-sectional areas of the PAS, when the symphysis region was reconstructed. Combined with the fact that symphysis reconstruction was always accompanied by a posterior repositioning, this is in line with the main result of the present study and emphasizes the need for more awareness of this matter. In both their and our study, however, the posterior movement did not significantly affect the total airway volume. Interestingly, they also found a significant effect of preoperative irradiation, which seemed to counteract the tendency for airway narrowing. In our study, conversely, the only significant effect of irradiation was a reduction in the volume of the nasopharyngeal airways.

Given all this and the many factors that differ from patient to patient individually, more research is needed to make conclusive statements. Clearly, creating ideal conditions for masticatory rehabilitation should remain a primary goal of mandibular reconstruction. However, the impact of the position of the graft on the anatomy of the upper airway should not be overlooked, as respiratory function is also highly relevant to the patient’s quality of life.

## Conclusion

The overall accuracy of mandibular reconstructions using FFF is high, but it still needs to be optimized. Research is therefore needed to identify problems and make improvements. Modern surgical tools focus on the bony reconstruction, which is important for masticatory rehabilitation. However, when positioning the FFF, the potential impact on the upper airway anatomy should also be considered in order to achieve adequate respiratory function. In the present study, a setback-like movement of the mandible was associated with a smaller hypopharyngeal minCSA. The influence of reconstructive surgery and graft positioning on the upper airway needs to become a focus of research. In addition to the anatomical changes, the clinical relevance of these changes needs to be further investigated, bearing in mind that the pathology and rehabilitation of reconstructive surgery patients is complex and multifactorial.

## Data Availability

Most of the generated and analyzed data is included in this published article. Detailed datasets of the current study are available from the corresponding author on reasonable request.
